# Why Do People Who Belong to the Same Clan Engage in the Same Entrepreneurial Activities?—A Case Study on the Influence of Clan Networks on the Content of Farmers’ Entrepreneurship

**DOI:** 10.3389/fpsyg.2022.873583

**Published:** 2022-06-02

**Authors:** Xiaoli Jiang, Xiao Ma, Zenian Li, Yongjin Guo, Anxin Xu, Xiaofeng Su

**Affiliations:** ^1^College of Marxism, Minjiang University, Fuzhou, China; ^2^College of Economics and Management, Fujian Agriculture and Forestry University, Fuzhou, China; ^3^College of Business Administration, Fujian Business University, Fuzhou, China

**Keywords:** clan network, relational contract, entrepreneurial learning, entrepreneurial ability, entrepreneurial motivation

## Abstract

Farmers’ entrepreneurship is a powerful breakthrough for solving the problems associated with “agriculture, rural areas and farmers.” Although studies have commonly used the same entrepreneurial activities to analyze farmers’ entrepreneurship, its deep economic roots have rarely been investigated. Investigating the internal development mechanism within the same industry is helpful for understanding farmers’ entrepreneurship motivation and decision making and is an important point at which to implement regional research and enrich the overall research on farmers’ entrepreneurship in the Chinese context. Based on a single-case study in Q Village, Fujian Province, this work identifies the key role played by relational contracts in entrepreneurship groups: reducing transaction costs, promoting investment in asset specificity, and improving contract flexibility. Moreover, this approach is conducive for different action groups in terms of stimulating entrepreneurial motivation in the initial entrepreneurship period and improving entrepreneurship learning ability in the long term. Primary Action Group transforms exploratory intuitive learning into exploratory compilation learning, and Secondary Action Group triggers the learning effect and makes a proprietary investment by utilizing intuitive formulaic learning and compiled formulaic learning, thus reducing unforeseen, contracting and verification costs. During the pattern maturity period, Primary Action Group rationally integrates the supply chain and forms a stable entrepreneurial paradigm, while Secondary Action Group does so to maintain prior information reserves and lower information search, supervised execution, and bargaining decision costs. The value cocreation ability of the same type of commercial modularity is formed, and the whole process of farmers’ entrepreneurship is completed. Our results have important implications for policymakers in China and other countries with clans.

## Introduction

At present, given the enormous number of farmers and limited urban ability to absorb the rural population, farmers’ entrepreneurship has become a powerful breakthrough for solving the problems associated with “agriculture, rural areas and farmers.” Establishing and supporting farm entrepreneurship have become a necessity to achieve sustainability and economic growth, especially rural economics (De Wolf et al., 2007; Naminse et al., 2019). Therefore, understanding the driving factors of farmer entrepreneurship is getting more and more attention. When putting the background into Chinese farmers’ entrepreneurship, what cannot be ignored are Chinese clan groups what are one of the most important social organizations in rural China (Greif and Tabellini, 2017). So, we specifically focus on the set of informal institutions that could affect members’ economic behavior, the more specific point is the clans, which are one of the most important vehicles of informal institutions in rural China.

In farmers’ entrepreneurship, it is common for members of the same clan to be mostly engaged in the same industry (TONGZONGTONGYE in Chinese and hereafter referred to as TZTY). For example, most villagers in a specific village in Jinjiang are engaged in the sacrificial paper business or ceramic business. Similarly, Chinese entrepreneurs who not only start businesses in China but also go abroad rely on blood and geographical overseas relationships. Not only do their country folk usually live in the same place but they also engage in very concentrated industries.

The academic community has conducted little research on TZTY. Strictly speaking, the network relationships involved in nonclan village entrepreneurship, such as that in Taobao Village, and clan village entrepreneurship are different, so their influencing mechanisms are thus different. Therefore, this research is focused on TZTY. In an environment where China’s market system is still not perfect and the business trust environment still needs to be strengthened, family relations have become the most ready-made, informal organizational resources for entrepreneurship. Low capital and labor costs are conducive to reducing transaction costs such as production, coordination, and trust costs in the early stage of entrepreneurship (Li, 2013). Clan and family networks help entrepreneurs reduce transaction costs and thus maintain enterprise operations. What is the influencing mechanism of clan networks on the homogeneous content of farmers’ entrepreneurship? This work suggests that clan members’ entrepreneurial contacts are centered on the pattern of the differential sequence network, based on home ethics, and then expanded outward; these members began to form acquaintance groups, thus developing relational rules, as experience is a long-term relational contract (Luo, 2011). Moreover, the general operation mechanism is the fair law of tool exchange. The foundation for clan members having the same career is a relational contract between them affecting their entrepreneurial decisions and the consistency of entrepreneurial content.

The manuscript aims to explain that why do people who belong to the same clan engage in the same entrepreneurial activities. Examining the internal development mechanism of TZTY is helpful for understanding farmers’ entrepreneurial motivation and decision making and is an important entry point at which to implement regional research and enrich the context of the research on farmers’ entrepreneurship in China. In addition, such an examination is helpful not only for improving the research on the social relations embedded in economic activities with Chinese characteristics but also for clarifying the influencing mechanism of farmers’ entrepreneurship within the clan network on the homogenization of entrepreneurship motivation, learning, and content.

We aim to contribute to filling these gaps by examining the influence mechanism of relational contract on TZTY. First, the study expands the research on institutional field of new institutional economics and analyzes the influence of informal institution on farmers’ entrepreneurial behavior. Taking the homogeneous reasons for commercial content to the entrepreneurial stage as the analysis level, this work finds that the informal system under the constraints of the resource endowment of entrepreneurial behavior influences internal laws and mechanisms and allows for the analysis of the interformation mechanism and the law between local society and the evolution of the market economy. Second, the dynamic constructionist relationships among the clan network, entrepreneurial learning, and entrepreneurial content homogenization are analyzed based on the internal mechanism. Third, apart from the previously used static research methods, we study the entrepreneurial learning process from a more dynamic perspective, focusing not only on the development of the clan network and farmers’ entrepreneurial ability in dynamic space but also on the intermediate process of the transformation of farmers’ clan social capital into entrepreneurial ability, expanding the boundaries of entrepreneurial learning theory.

The outline of the paper is as follows: Section “Literature Review and Research Framework” and “Research Design,” we demonstrate our method, case selection, data collection, identification and description of key constructs. Section “Case Analysis and Research Findings” and “Conclusion and Implications.” We present the followings.

## Literature Review and Research Framework

### Literature Review

A promising and fruitful line of research seeks to explain the definition and influencing mechanism of Chinese clan groups. The clan groups are a population that was bred from the same ancestor, usually linked by common property and weddings and funerals, and lives in the same village. Since clans exist in many places, especially Asian countries such as Singapore (Freedman, 1958), Korea (Yang, 2019), and Philippines (Cruz et al., 2020), the study has general implications beyond China.

Entrepreneurial activities have shown a significant impact on improving the standard of living, especially the farm business of rural people (Naminse et al., 2019). Many scholars have argued that high-quality entrepreneurial networks help entrepreneurs identify potential opportunities (Davidsson, 2015). Yu et al. (2021) found that heterogeneous networks are positively related to innovative opportunities, and homogeneous networks are positively related to imitative opportunities. Clans are highly important institutions since they facilitate cooperation (Greif and Tabellini, 2017). Clans have networks that are formed by blood relationships, which may share job information with unemployed members or help them find jobs directly (Foltz et al., 2020). The clan networks may also help members overcome financial constraints and establish businesses (Kinnan and Townsend, 2012; Zhang, 2020). Clans have positive effects on male entrepreneurship but no effects on female entrepreneurship (Li and Huang, 2021). At present, the research on the influence of clan networks on farmers’ entrepreneurship is not abundant enough, while that on the homogeneity of clan networks on farmers’ entrepreneurship in the same industry is even less.

However, some studies have taken a peer research approach, considering traditional peer performance in society as either regional or ethnic business. For example, Gao (2006) found a relationship between peers and their hometown, while Zheng (2014) noted that a key factor in peer relationships is the credit between private enterprises relying on the local social and cultural network. All-in-one industry is a rural development model that integrates people, townships, and industries (Sun and Li, 2020).

The main explanation for the “fellow industry” phenomenon is as follows. Tan (2012, 2020) analyzed cultural intimacy as the core mechanism behind the “fellow hometown” from the perspective of kinship, geography, and market interaction to help enterprises gather resources, reduce costs, and win market competition. Furthermore, Wu (2020) suggested that “peers,” as a form of social and economic development, depend on whether the economic activities of a certain scale can involve the appropriate industries at the appropriate time through the social network built by traditional society. This whole process reflects the interaction between the economy and society.

There is even less research on the homogeneity of the content of farmers’ entrepreneurship; thus, it is necessary to clarify its internal mechanism, understand its limitations and contribution in the process of urbanization, and provide more formats for innovative entrepreneurship to subsequent farmers, thus also providing certain policy advice. Because the entrepreneurial stage of farmers is small scale and high risk and because the external market is not a perfect capital market and support system, family enterprises often find it difficult to obtain foreign investment. Familiar financing can overcome obstacles such as information asymmetry and, at the same time, has the advantage of low cost and provides important financial support to family enterprises (Han et al., 2014). With the support of rural social networks, farmers can obtain the funds, information, and resources needed to start a business, thus improving their willingness to start a business (Zhang and Feng, 2019). Then, in future entrepreneurial activities, diffusion, clustering, and growth exhibit the typical characteristics of the kinship drive (Ma and Yang, 2011).

In conclusion, there is little research on clan networks and TZTY. The first factor that should be considered in such research is the mechanism behind the clan network. Second, some scholars have paid attention to the construction of family business models, but most are empirical in nature, rather than systematic, and do not focus on the cooperative evolution of entrepreneurial motivation, learning, and business models under the clan network. The existing research also does not analyze the relationship between the clan network and the development of farmers’ entrepreneurial ability from a more dynamic perspective. How is the social capital of the individual clan network transformed into the intermediate process of entrepreneurial ability? How can the clan network be activated and farmers’ entrepreneurial motivation be promoted? How can clan members gain unique entrepreneurial ability through entrepreneurial learning? How can the homogeneity of entrepreneurial behavior and content be improved?

Goetz and Scott (1981) defined a relational contract as a contract that cannot be reduced to well-defined obligations. This paper agrees that a relational contract is a form of contract implemented by an individual through a long-term repeated game (Wang and Li, 2008), also known as an implicit contract or informal system, and is not shown through explicit text during implementation. A maintenance relational contract requires only the contract party to know local information about the relevant contract variables and to have the same expectation for the results. Internal long-term games lead to short-term opportunistic losses because the cheater in the game loses all his or her future expected gains or cooperative surplus and exhibits self-implementation, thus ruling out reverse selection and locking in moral risk (Kranton, 1996). Subsequent studies discussed the details of relational contracts. Aghion and Holden (2011) provide an overview of incomplete contracts and refer to relational contracts as part of second-generation models of incomplete contracts. Gil and Zanarone (2018) review mostly empirical research. Rigorous case studies of relational contract theory are still in its infancy.

A relational contract (Sun and Yu, 2010) has the following characteristics: relational embedding, extended duration, self-enforcing, and open terms. Subsequently, Wang (2006) constructed connected relational contract theory and expanded the concept of traditional single-market relational contracts into connected relational contracts. The relational contract can formalize the idea that increased trust can make relationships more productive (Michler and Wu, 2020).

Long-term and incomplete transaction arrangements define the future rights and obligations of relevant parties when they value their transaction relationship (Sun, 2013). The implementation mechanism of this relationship constitutes an appropriate trading framework that often includes various procedures, commitments, rights, incentives, trust and emotions, and relational contracts can undoubtedly reduce transaction costs. At the same time, relational contracts can motivate people to carry out informal cooperation, and by partially adjusting and/or creating incentive and coordination mechanisms, formal governance can maintain relational contracts within its “self-executing scope” (Baker et al., 1994, 2002; Argyres et al., 2020). In other words, a relational contract can replace a formal contract when the social and legal systems are not perfect, stimulate proprietary investment when transaction details cannot be verified, and realize governance by using relational rules to reduce transaction costs.

In summary, a relational contract is based on the limited rationality of the contract party and the existence of transaction costs, which leave room for improvement in the contract in terms of continuous negotiation in the relational system (Liu, 2003). Simple examples, such as the use of resources by rural enterprises, are based only on the legal system, while other more complex examples are embedded in the personalized relational system (Liu, 1999). Relational contracts are implemented essentially by individuals through long-term repeated games, where the trust formed during past transactions and other relational rules promote their implementation for the purpose of achieving aggregate point equilibrium (Li, 2003).

### Research Framework

How can a relational contract explain this phenomenon by solving two main problems: whether the relational contract exists in the clan network and how it affects the homogeneity of the entrepreneurial model of clan members? Relational contracts are prevalent in the Chinese context, especially in villages with clan networks. Guo (2011) suggested that the relational contract between a contractor and “migrant workers” is a contract of even if there is no written contract, mutual responsibilities and obligations, similar to the trade rules, exist and, thus, can be considered a valid contract in terms of performance. Yang (2007) found that the cooperation mechanism of the Wenzhou Chamber of Commerce in the initial stage was mainly a relational contract. Moreover, Luo (2011) suggested that Chinese enterprises are often embedded in business clusters and industrial networks. To broaden relational resources, actors need to exchange with others and establish relational contracts to create long-term human exchanges. Luo (2017) found that both incomplete and relational contracts exist for Chinese agricultural land transfer leases, considering the relational contract as an important mechanism with which to eliminate the costs of incomplete contract management. An intermediary, formed by acquaintances, between strong and weak companies is also a major feature of Chinese behavioral decisions, showing that in China, relational and formal contracts coexist.

The relational contract between clan members is manifested mainly in the construction and performance of the trust mechanism in the entrepreneurship process. First, no formal and complete written contract is signed between partners. The cultural rules in the traditional clan network stipulate the behavioral code of organizational members when an unforeseen situation occurs. That is, through the regulation of the behavioral rules system, cultural rules can replace complete formal contracts (Williamson, 1996). Here, cultural rules mean that the cooperation norms in the kinship network encourage network members to cooperate by improving the distribution of rights, reducing the careless behavior of individuals, and overcoming the hitchhiking phenomenon. Second, in relational contracts, partners are convinced that the other party has the ability and faith to cooperate to complete tasks. Moreover, expectations for a long-term future contract make both parties more confident in terms of investing in this partnership. Third, compared with economic interests and operational risks, partners view this partnership very optimistically (Uzzi, 1997). Although no individual in the clan network can accurately predict the future development trend of entrepreneurship, with the trust and human relationships within the network of acquaintances, individuals can independently mediate and solve problems to a certain extent. Therefore, cooperation is based more on default informal rules, that is, relational contracts.

Relational contracts in stimulating entrepreneurial motivation, improving entrepreneurial learning ability, and promoting business model homogeneity play an important role. Learning behavior is a key source of building entrepreneurial capabilities (Futterer et al., 2018), and individual learning research has always been the focus of scholars in entrepreneurial contexts (Tseng, 2013).

Despite the diverse and intersecting perspectives, few studies have been able to deeply deconstruct learning as a process (Johannisson and Huse, 2000; Cai and Shan, 2013). Drawing on the individual learning framework proposed by Politis (2005), this paper argues that entrepreneurs can transform experience into two types of key entrepreneurial knowledge. Among them, the first category is related to opportunity identification—there are two paths: (1) there are prior information sources needed to identify opportunities in experience, and the number of information sources will determine opportunities the size of the recognition ability (Jones and Casulli, 2014); (2) the continuous accumulation of personal experience and timely reflection will dynamically affect the individual’s ability to assess the value of opportunities—the cognitive basis for identifying means-result relationships (Shepherd and Patzelt, 2018), while the second category is related to dealing with the difficulties of entrepreneurship, which means that individuals acquire knowledge about business skills, network relationships, and reputation through learning from previous experience, which are used for processing including finding capital, building legitimacy, and acquiring social networks (Politis, 2005). Therefore, the focus of individual learning is how to transform experience into entrepreneurial knowledge, and this process will determine the choice of learning methods and the corresponding learning effects.

Based on the perspective of experiential learning theory and process, and referring to Chen et al. (2020), the dual classification of exploratory transformation and exploitative transformation for individual learning, two different learning modes (exploratory and exploitative), determines the formation of different entrepreneurial knowledge. The former focuses on acquiring and improving the knowledge and ability of entrepreneurial opportunity identification effectiveness through experience transformation; while the latter emphasizes obtaining knowledge through the transformation of other people’s experience to improve the ability to deal with new entrepreneurial difficulties.

Relational contract can work in the following three aspects. First, it manifested mainly in the form of relational property rights (i.e., consensus in the relational transactions concerning property status) for the individual and in the form of individual relational interactions, thus reliably committing to the contract, which is conducive to the investment of dedicated assets. This relationship is a short and specific social network relationship, which is based on the relational contract concerning property rights, can be used as a channel through which to obtain entrepreneurial resources, and is also a strategic countermeasure taken by individuals to establish stable channels through which to obtain relevant resources and entrepreneurial opportunities or to reduce risk.

Second, a relational contract in the clan network group concerned with entrepreneurship can reduce transaction costs, such as those for the order procurement form, sharing operations, and other key information, reduce the threshold entry and procurement costs, alleviate venture capital constraints, and reduce market search costs, as well as the risks associated with entrepreneurship. At the same time, such a contract promotes the asset-specific investment of entrepreneurs and lays the foundation for the homogeneity of the subsequent entrepreneurial business model.

Third, the relational contract has high elasticity, which is conducive to the two parties adopting cooperation and other compensatory technical means to deal with possible differences and is also an important mechanism through which to reduce the cost of inefficient contract governance. Compared with the low elasticity of entrepreneurial content, individuals are more willing to engage in an entrepreneurial business model with high elasticity and low risk.

## Research Design

### Research Methods and Case Selection

#### Research Methods

From the perspective of the research object, TZTY is a construct with contextualized characteristics, and case study is a more appropriate research method to explore contextualization (Welch et al., 2011). The reasons for the use of the single-case analysis method in this study are as follows. First, the case study approach is applicable for exploring the problems related to how, process and mechanism, can help refine the theory or laws behind a phenomenon and can effectively demonstrate the integrity and dynamics of the research process. The purpose of this paper is to analyze the “what” and “how” mechanisms. In addition, because the whole entrepreneurship process is multistage and co-evolutionary, the use of single-case longitudinal research is conducive to clarifying the reasons for and process of the learning interaction mechanism of different behavior subjects and the reasons for their influence on innovation behavior and that of clan networks on farmers’ entrepreneurial business model from the “process” perspective (Eisenhardt, 1989).

#### Case Selection

According to the principle of selecting the most suitable sample cases, this paper sets two case selection criteria: first, the village must belong to a clan network in which all members have the same surname, with an ancestral temple or family tree in the village, and second, most villagers carry out the same entrepreneurial activities. Based on the above reasons, Q Village in Yongtai County, Fujian Province, was selected as the case study object. The team conducted research and observations in the village from September to October 2019 and July 2020 and accumulated a large amount of data. A brief introduction of the situation in Q Village, the development of its clan network, and the situation of villagers’ entrepreneurship is presented below.

Q Village now has more than 1,200 members, with a total area of 9.6 square kilometers. The village elevation is 610 meters. The whole village is surrounded by 8-kilometer-high peaks. The village is rich in natural ecological resources, with more than 8,000 Mu of ecological forest area and more than 3,000 Mu of land being part of Qinggang Forest Protection Reserve in Fujian Province. Before a road was built, traffic in the village was very underdeveloped. In the era of industrial civilization, the village was considered a typical village, with weak resource endowment. Q Village is a typical family village, and the surname of its villagers is Chen, which belongs to the Yingchuan Chen family (Henan Province). Older people in the village generally have a genealogy that can be traced. In 2016, villagers raised more than 2 million yuan to rebuild the Chen Ancestral Temple according to its original layout, a task that was completed in 98 days.

**Figure 1:**
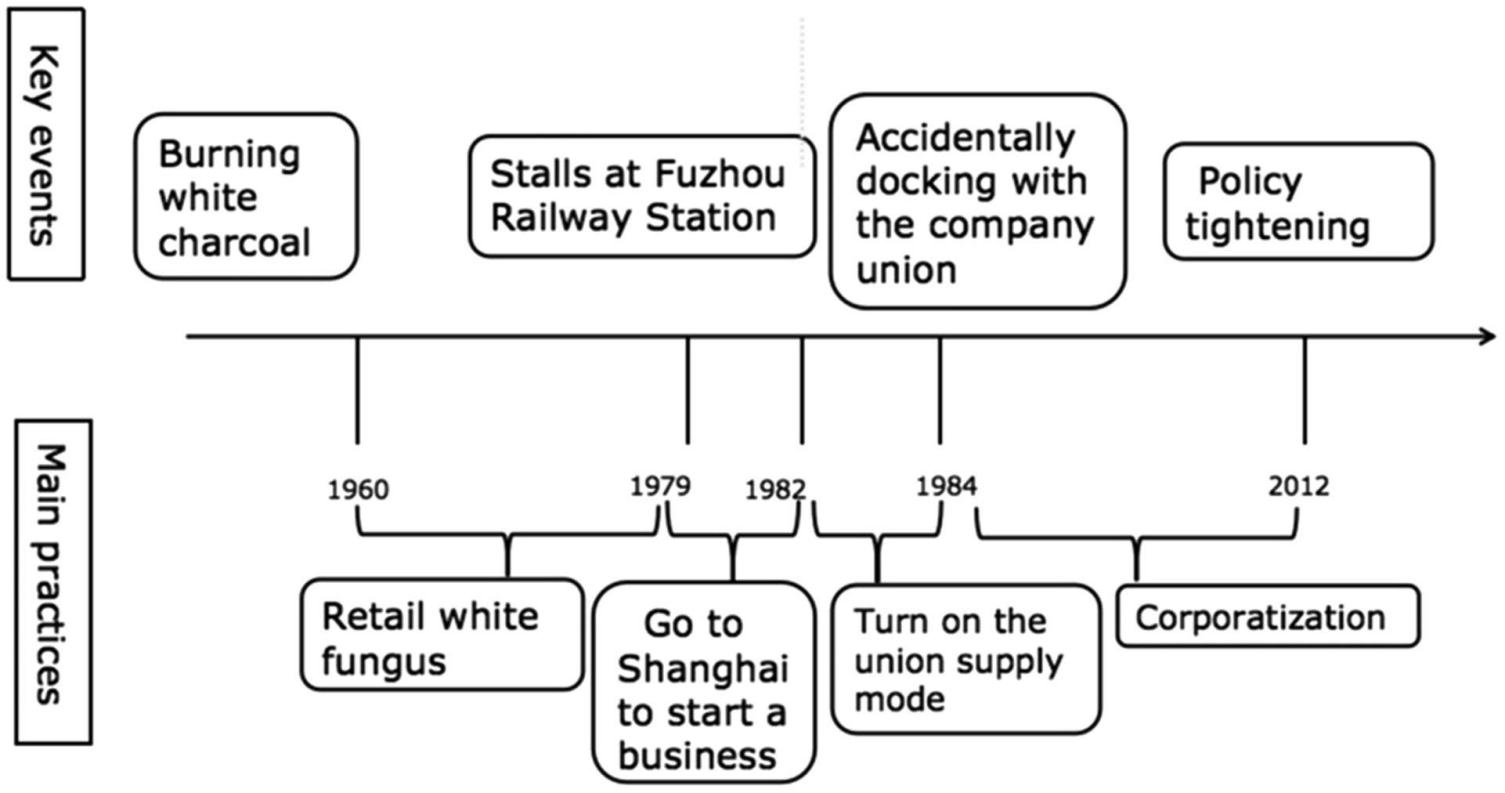
Key events and main practices in Q Village since 1960.

The entrepreneurial history of villagers in Q Village can be divided into three stages. In the 1960s, white charcoal was burned in the area; in the 1970s-80s, Fuzhou Railway Station went through a stage in which it sold white fungus; and in the 1980s, some villagers went to Shanghai to sell white fungus and other dry cargo (see Figure 1).

### Data Collection

#### Data Source and Collection Method

In October 2019, the author participated in a field workshop with Q Village as the main body. More than 20 people lived in the village at that time. The village situation and rural governance were investigated, and the entrepreneurial situation of villagers whose team members went to Shanghai in May 2019 was taken as the basic study topic. From July 1 to July 31, 2020, the village committee planned to compile a history of Q Village, attracting more than 10 volunteers to share their oral history. As one of the volunteers, the author investigated and collected mainly the entrepreneurial information of the villagers. Most of the field workshop team and oral history volunteers had bachelor’s degrees or above, with different age structures, geocultures, and educational backgrounds, thus reducing the possibility of homologous variance.

During the research process, the snowball method was used first to investigate the village committee; then, the village committee was asked to identify villagers who went to Shanghai to start a business. Next, the committee was asked to identify those villagers that went to Shanghai in the second and third waves according to our requirements. Table 1 shows the interview data.

**Table 1:** Data collection methods.

Data type	Source	Theme	Detailed description
In-depth interview	Village committee	History of village development	Multiple rounds of in-depth interviews included more than 10 entrepreneurs in Q Village in the form of semistructured interviews; 10 people were interviewed 18 times, at a duration of 700 min
	Village entrepreneur	Entrepreneurship history and details	
Field observation	Village history writing mobilization committee	Mobilization process, suggestions for and expectations of entrepreneurs on the development of the clan and village	In the mobilization meeting of Q Village’s history compilation committee, speakers were township government representatives, clan representatives, and enterprise representatives; they observed the relationship between villagers, etc.
Internal documentation	Villagers	Some archives in the process of starting a business	Clan genealogy, entrepreneurial data, etc.
External second-hand documentation	Internet	News report	For example, the news reports on villagers going to Shanghai to start businesses

#### Semistructured Interview Data

Davis and North (1992) pointed out that in institutional innovation, the subject of action can be divided into Primary Action Group and Secondary Action Group. We follow this paradigm, the selection criteria for interviewees are as follows: (1) Village Entrepreneurship Primary Action Group can provide an overall timing perspective for this study; (2) villagers who directly participate in entrepreneurship can provide first-hand information and triangular verification of the key information of the former interview; and (3) age levels cover three generations, including Secondary Action Group, which can help achieve a rich research perspective. Eventually, 10 people were interviewed more than 18 times.

The interview outline includes two main parts: the first part is an open interview, including the interviewer’s life experience, entrepreneurial experience, and business model, and the second part focuses on the interviewee’s entrepreneurial experience in Shanghai. When talking about his business model, we suggest that the interviewer tell the story behind it and conduct cross-validation.

### Identification and Description of Key Constructs

#### Encoding Process

Interview records were coded and theoretically saturated; then, the data were analyzed according to programmed root theory suggested by Strauss, namely, open-ended coding—spindle coding—selection coding. To ensure research reliability and validity, we performed data collection as well as initial data analyses by trying to figure out patterns in the data (data coding and development of first order concepts and their organization into second-order themes) simultaneously. Through the simultaneous data collection, coding, and initial analyses, our goal was to identify any new conceptual ideas. Moreover, school entrepreneurial experts were consulted, and then, data were modified and deleted accordingly, to avoid the coder being too subjective, and improve the objective reliability of the coding.

Open-ended encoding was performed first. Data mining was carried out with existing text data, as paragraphs and chapters unrelated to this study needed to be excluded before coding. To reduce the perceptual influence of individual researchers, we used the text and original statement as labels as much as possible to explore the initial concept and summarize all 33 First-Order Concepts. Some of these statements could generalize multiple concepts. By comparing and refining these scattered concepts, the relevant concepts were integrated to realize the dimension, and finally, four aggregate dimensions were formed.

Second, spindle coding was carried out. This step was taken mainly to establish various relationships between categories to represent the organic associations between various data parts. According to the steps of spindle coding, through open-ended encoding to better develop the main category, we found that the different categories obtained in the open-ended encoding had certain internal relations, according to the mutual relational contract between different dimensions. Furthermore, the open-ended encoding dimension and dimension connotation corresponding to each main dimension were clarified (Wang, 2019).

We reached saturation when no additional data to develop new themes were found in subsequent interviews. Then, we assumed that we have the full set of first-order concepts and second-order topics as a basis for developing aggregate dimensions and building data structure. Finally, selective coding was performed. The main dimensions and its logical correlation were determined, and the various dimensions were systematically integrated by choosing the core category to build the theoretical model. The core dimensions identified in this study were the homogeneous business model of clan members, and the basic relational contract ran through the whole process of farmers’ entrepreneurship. This paper divides the entrepreneurial process of the case enterprise into the following three stages: the initial entrepreneurship period, the “crossroads” period, and the final entrepreneurial period. The classification basis mainly considers that there are differences between environmental uncertainty perceived by entrepreneurs at different stages and resource constraint level of the organization (Hao et al., 2019; Chen et al., 2020). Specifically, in the entrepreneurial initial period, the “crossroads” period, and the final entrepreneurial period, clan network members, because of blood, geographic, and industrial relations, can be categorized into Primary Action Group and Secondary Action Group in terms of entrepreneurial motivation, entrepreneurial learning, and entrepreneurial cognitive process, respectively, to construct the theoretical model.

#### Key Configuration Point Identification and Description

Through data coding, four key dimensions are generated, namely, entrepreneurial motivation stimulation, entrepreneurial ability learning, entrepreneurial model homogeneity, and relational contracts. Business model content addresses the premise and fundamental problems of the logic behind business model value creation (Martins et al., 2015). The main reason for Q Village’s business model being formed to supply dry goods of the trade union system was that the endogenous relational contract in this clan network was a result of villagers’ decision making, including their decisions regarding entering businesses and the entrepreneurial business model. In this study, it was first necessary to distinguish the action groups that entered the market at different times and with different incomes and action strategies. Accordingly, this chapter divides Q Village into those members who started businesses in Shanghai—Primary Action Group—and those in the groups that followed suit—Secondary Action Group. According to the interview data, we analyzed how the relational contract affected the entrepreneurial behavior of different action groups, including how it stimulated entrepreneurial motivation, improved entrepreneurial learning ability, and cognitive mode, and finally led to the homogenization of the entrepreneurial business model, that is, the phenomenon of members being in the same industry (Table 2).

**Table 2:** Data structure.

1st order concepts	2nd order themes	Aggregate dimensions
Survival needs m11	Survival entrepreneurial motivation M1	Entrepreneurial motivation to stimulate (M)
Human-land tension m12		
Improved life m21	Developmental entrepreneurial motivation M2	
Market environment m22		
Share supply channel m31	Share entrepreneurship information M3	
Share sales m32		
Startup funds available within the clan network m41	Activate production factors M4	
Product can be produced m42		
Labor is repriced m43		
The unknown is explored c11	Exploratory intuitive learning C1	Learning entrepreneurial ability (C)
Previous information is restored c12		
Product has operating advantage product c13		
Good at marketing c21	Exploratory compilation learning C2	
Good at discovering opportunities c22		
Mimic peers’ entrepreneurship c31	Exploitive intuition-based learning C3	
Learn with existing experience c32		
Take action while practicing c41	Exploitive compilation learning C4	
Follow relatives to find business c42		
Group entrepreneurship p11	Entrepreneurship content design P1	Entrepreneurship content is homogeneous (P)
Internalized behavioral norms p12		
Asset-light operation p21	Homogeneous of entrepreneurship content P2	
Form business model p22		
Integrate supply chain p31	Team behavioral integration P3	
Integrate human capital p32		
Internalize external risk p41	Value cocreation capability P4	
Peer-propagation effect p42		
Unforeseen contract costs g11	Reduced transaction costs G1	Relational contract (G)
Concluding costs of contract g12		
Confirmation costs of contract g13		
Structured transaction g21	Contract elasticity G2	
Reasonable rule g22		
Internalization reduces external risk g31	Asset specificity G3	
Asset specificity g32		

## Case Analysis and Research Findings

### Relational Contracts Stimulate Entrepreneurial Motivation in the Initial Period

From the end of the 1920s to the end of the 1970s, Q Village was located in a mountainous area, taking a typical “eight mountains, one water, one field” form. In the 1960s, many villagers relied on burning and selling white charcoal, but in later years, this trend gradually declined, and villagers had to rely only on farming to make a living. For example, one villager, CJL, cannot engage in heavy farm work due to physical reasons and, thus, to make a living, must buy white fungus and then sell it at Fuzhou Railway Station. His nephew, CYT, sees his being bullied by other regional vendors in the railway station; then, CYT follows his uncle into business so that he can make sure that no outsiders bully his uncle. The initial entrepreneurship of Primary Action Group is given priority but is limited by local tensions; members of this group need to change their living environment, but entrepreneurial content is fully dependent on local resources, such as white fungus. Moreover, within the same clan network, group members have developed deeply rooted habits. At this time, the search for scientific knowledge promotes exploratory management. In the late 1970s, when the sales environment at Fuzhou Railway Station became increasingly difficult, CYT’s friends in Fuzhou recommended that he and his uncle start their own business in Shanghai. These friends believed that Shanghai provided great opportunities in terms of market demand that its business environment was relatively formal, thus eliminating the phenomenon of being bullied by local vendors. CYT thought twice about boarding the train to Shanghai but also opened the people of Q Village to the idea of conducting business in Shanghai.

Experience is a source that is required to identify opportunities, the amount of which will determine the magnitude of opportunity identification (Jones and Casulli, 2014). Members of Primary Action Group went to Shanghai from the stalls and continued to sell the same resource—white fungus. At this time, other contemporaries also went to Shanghai. Due to the reform and opening up in China, market demand increased, and Q Villagers were able to sell white fungus inexpensively. For the members of Primary Action Group, heading to Shanghai to pursue entrepreneurship was both bumpy and smooth; there was more than that of the villagers who stayed in Q Village to farm. For example, in 1980, CWJ made “5,000 yuan per month in Shanghai” and could “earn a month’s salary of an ordinary worker in a short time by selling dozens of pieces of white fungus. For instance, by selling 50 packages (of white fungus), I earned at least 100 yuan.” In Secondary Action Group, members earned social income, produced by originally idle or half idle labor, and achieved capital pricing, originally local investment income and farming income, which are obviously not from conducting business in Shanghai; material interests inspired these members to carry out entrepreneurship. However, at this time, many members are facing constraints in terms of initial entrepreneurial capital, and thus, the family plays an important role in providing low-cost financing within the network. An example of such financing was provided by CYT, who “borrowed 100 yuan” and whose uncle also promised to give him 100 yuan. Such an example is not the only one in this case; another example can be seen in the case of a clothing founder buying raw material cloth, making up for its lack of funds through familiar financing.

At this time, the relational contract in the clan network is reflected mainly in core information sharing among members and has advantages in terms of obtaining key resources, providing convenient channels for resource mobilization and transfer among members. Moreover, there is a law of demand regarding acquaintances; that is, as long as they are considered part of the same circle, then they share the same benefits. Mutual transactions between acquaintances and friends are act as a type of self-reinforcing equilibrium (Kranton, 1996). Chinese people find a sense of belonging in their idea of “everyone” and a sense of achievement in their own “small home.” This sense of achievement includes telling their peers about the outside world as well as their personal successes there. Peers share two types of knowledge: declarative and procedural. In general, declarative knowledge includes concepts, values, general beliefs, and secular beliefs about the self, other people, and the social world. Procedural knowledge includes tricks, behavioral scripts, practices, and routine processes. Action Group I shares mostly procedural knowledge, that is, the details of starting a business in Shanghai, including the sharing of the sales information of the supply market, details concerning to which market it is better to sell, and where to buy wrapping paper. Secondary Action Group went to Shanghai in the 1980s and 1990s, for both survival and opportunistic reasons, such as entrepreneurial motivation; some villagers were indeed forced by life circumstances to do business there, while others saw the opportunities for development there. At the same time, the stability brought about by the expectations of relational contracts also enabled entrepreneurs to achieve opportunistic entrepreneurship. Table 3 lists examples of illustrative quotations in the initial entrepreneurial period.

**Table 3:** Illustrative quotations of first-order concepts in the initial entrepreneurship period.

Second-order theme	First-order concept	Illustrative quotations
Survival entrepreneurial motivation M1	Survival need m11	“My family of eight on 7–8 plots of land… When I was free, I sawed boards outside and earned 100 yuan; then, I came back home to plant sweet potato and took the money back to buy salt, children’s clothes and so on, all of which relied on the 100 yuan.” Am1
	Human-land tension m12	“The village is all in the mountains. We could not make a living by burning white coal before… Then, we went back to the farm and burned charcoal for 50 or 60 days.” Bm2
Developmental entrepreneurial motivation M2	Improved life m21	“Looking at everyone else going to Shanghai, there are old people and children at home; I tried my hand at it.” Cm3
	Market environment m22	“At that time, material was scarce, and there was no money to buy. There was no place to buy shiitake mushrooms and longan.” Bm5
Share entrepreneurship information M3	Share supply channel m31	“At the beginning of local production, later, we went to Gutian to purchase more than just white fungus.” Am1
	Share sales m32	“So, the reputation came out, and everyone knew to go to Shanghai to sell white fungus.” Cm3
Activate factors of production M4	Startup funds available within the clan network m41	“I borrowed 100 yuan from my father-in-law, and at that time, 100 yuan was a lot. My uncle also promised to give me 100 yuan.” Dm4
	Product can be production m42	“In 1979, fungi were first planted in Gutian County; so, we ran outside to collect the fungus.” Em1
	Labor is repriced m43	“I used to work outside for more than half a year to earn 100 yuan. Now, I sell 50 kg of white fungus in Shanghai and earn 1,000 yuan.” Am1

*Based on this, propositions A1 and A2 are presented*.

**Table 4:** Illustrative quotations of first-order concepts in the “crossroads” period.

Second-order theme	First-order concept	Illustrative quotations
Exploratory intuitive learning C1	Explore the unknown c11	“At that time, I was timid, bought the ticket, and got things packed but got the refund twice, so I dared not go. At that time, for example, I had only 1,000 yuan; I was afraid of being robbed and could not survive. After a long time of mind struggle, I still went to Shanghai.” Ac1
	Restore previous information c12	“(White fungus) sold to passengers, who always took some white fungus home.” Bc1
	Product has an operating advantage c13	“At that time, there were mushrooms and longan. The union staff said that you have only white fungus, and you can sell longan. Longan stacked into box, like mahjong. Sometimes, we sold 8,000 kg, earning 10 yuan per kg.” Ac1
Exploratory compilation learning C2	Good at marketing c21	“Our white fungi are inexpensive and good.” Bm2 “The union staff helped me to sell; he got 50 cents from a package. Some may not accept money, so I send something to them.” Dc5
	Good at discovering opportunities c22	“After being sold, we thought that the stall could sell only 2.5 kg a day. He (the chairperson of the trade union) suddenly sold 25 kg, and not at a short weight, but the price was more expensive than that in the market. In the market, I negotiated a price of approximately 25 yuan, and he (the customer) said 18 yuan, 20 yuan, 22/23 yuan, and now, he (the chairperson of the union) agreed with 25 yuan. Later, I said we do not go to the stall, and we ask to see the factory.” Ac2
*Exploitive* intuition-based learning C3	Mimic peers’ entrepreneurship c31	“I have taught people of the same clan what department to look for. Get down to talking about business.” Ec1
	Learn with existing experience c32	“After 1985, I sold shiitake mushrooms. After 1983, I bought white shiitake fungus in Gutian, the price of which was a few cents higher than that in the countryside. The people of each group bought their own white fungus, CCZ did the accounting in Gutian, and then, we jointly set off from Gutian to Shanghai.” Fc1
*Exploitive* compilation learning C4	Take action while practicing c41	“We had a good division of labor; one was responsible for retail, the other was responsible for acquisition, and the family model was united. We all contacted company, not stalls. It was very easy to get in contact at that time; I sent something to the chairperson of the trade union, and our goods from the countryside were more real.” Ec2
	Follow relatives to find business c42	“When there was difficulty, most people turned to CYT (the first one to conduct business in Shanghai) for help. In the second half of 1982, I took my cousins and our villagers to Shanghai. Later, CYT engaged in wholesale in Maojiatang, and we took the goods directly from there.” Fc2 “Take relatives, convenient business.” Cc 2

*Based on the above, propositions B1 and B2 are presented*.

Based on this, propositions A1 and A2 are presented.

Propositions A1: *In the initial entrepreneurship period, the relational contract formed through interactions among clan network members helped the members of Primary Action Group share its procedural knowledge of entrepreneurship*.

Propositions A1: *In the initial entrepreneurship period, the exploratory transformation of Secondary Action Group helped activate the potential elements of entrepreneurship (such as labor and capital), thus stimulating entrepreneurial motivation*.

### Relational Contracts to Promote Entrepreneurial Learning in the “Crossroads” Period

In the “crossroads” period, individuals are faced with a series of decisions regarding whether and how to start a business. Entrepreneurship is essentially a learning process. The learning mechanism is an important means through which to maintain superior resources, and the key indicator is whether entrepreneurial learning ability is acquired in a smooth manner.

For Primary Action Group entrepreneurs, information collection and processing costs required certain human capital investments, including but not limited to investing much time and energy in identifying entrepreneurial opportunities, analyzing entrepreneurial risk, identifying entrepreneurial partners, developing business plans, and implementing entrepreneurial activities. Each link behind the information collection and processing costs constitutes the entrepreneurship threshold. When members of Action Group I determined the entrepreneurial content, they continued to deal in local products, given their entrepreneurial experience, and transformed them into a form of exploratory compilation learning on the basis of exploratory intuitive learning. Their main purpose was to continue to explore opportunities for discovery. By chance, Action Group I members encountered a trade union chairperson who could buy all their goods; however, after considering the income that they would earn, they decided to pursue the opportunity, a form of exploratory compilation learning.

Secondary Action Group initially utilized intuitive learning, which means the use of an existing empirical model and the imitation of Primary Action Group. In general, people who belong to social networks act rationally when facing new environments, but once institutionalized balanced behavior is self-formed, individuals’ optimal action strategy is imitation. Entrepreneurs deliberately practice imitation to improve their performance (Baron and Henry, 2010). Two types of learning models are recommended in psychology to account for imitator dynamics: one type is a social learning model, and the other is a stimulus–response learning model (Fredenberg and Xiao, 2004). For members of the clan network with these two kinds of learning models, they have more options with the expanding market and can do business everywhere. This case is not an exception, as in Fuzhou, people do business overseas, but some enterprises cannot be directly reached; thus, these entrepreneurs spend time in their hometown shops or enterprises as apprentices to gain experience. For example, as one member stated, “After having certain experience in other people’s enterprises, I learned to have a set of rules and systems of modern management to adapt to the market rules, and I just came out to do business or run enterprises independently.”

Moreover, there are also cooperative and competitive relations between clan members, as they all are hoping to start a successful business in Shanghai. This stage requires a combination of exploitive compilation learning, that is, taking action on the basis of imitation. For example, CDT watched his predecessors win the business of the health system union, and he believes that the business of the school system also has great potential, so he tries to find opportunities to finally pursue the relevant business of the school system.

The relational contract plays a main role in the relationships between the entrepreneurs of different action groups, reducing the transaction costs of both parties and guiding the group in making cumulative and asset-specific investments. Collective clan behavior reduces transaction costs, which include mainly economic transaction costs, social communication costs, and organizational mobilization costs. There is transaction fee because information comes at a cost, and the distribution of information between exchange parties is asymmetric. Innovation in reducing transaction costs is composed of innovations in organizations, tools, specific techniques, and implementation features. These innovations can have one of three cost margins: to increase capital liquidity, to reduce information costs, and to spread risk. In the clan network, the latter two are particularly obvious, manifested in a high degree of entrepreneurial information sharing and low asymmetry among members; the clan network has an internal ability to handle external risks. These functions internally disperse entrepreneurial risks and thus increase clan members’ risk preference.

For Primary Action Group, available internal and external resources need to be invoked quickly at a low cost to ensure survival (Han et al., 2014). Group entrepreneurship is conducive to resisting the competitive pressure of other regional groups. At the same time, the number of entrepreneurs is large, so order procurement can be used to reduce procurement costs. In many relational networks, family ties are seen as the foundations of an emotional relationship, and the ethical obligations in such relationships contain less instrumental meaning than do those in other relationships. However, in fact, the importance of family instrumental relationships should not be underestimated. With the development of the market economy, members have emotional contracts and tool contracts in terms of their actions; that is, mutual help is aimed at both out of emotion and out of interests. Following the group, due to the previous exploration of Primary Action Group, we can clearly realize the entrepreneurial path, save market search costs as well as a series of costs, such as foreseen costs, contracting costs, and confirmation costs, and reduce entrepreneurial risks. If the value of future cooperation is large enough, then relational contracts can stimulate proprietary investment. Members of Secondary Action Group, after estimating their expected future income, accumulated asset-specific knowledge and investment; that is, they fully understood the details of entrepreneurial activities and invested capital in the purchase of entrepreneurial goods.

Propositions B1: *In the “crossroads” period, Primary Action Group transformed exploratory intuitive learning into exploratory compilation learning, which combines emotional and tool-based relational contracts to reduce transaction costs*.

Propositions B2: *In the “crossroads” period, Secondary Action Group triggered the learning effect through exploitive intuitive learning and exploitive compilation learning, reduced forecasting costs, contracting costs and confirmation costs, and then made proprietary investments*.

### Relational Contracts in the Final Entrepreneurship Period

In particular, most clan network members start their own businesses in Shanghai (far away from the familiar living environment of their hometown), in which the relational contract between clan members is a joint “blood relationship” and “geography,” implying the concept of an internal closed loop, transaction frequency, trust mechanism, and boundary. As mentioned above, group entrepreneurship can help entrepreneurs resist risks in an unfamiliar business environment and reduce their financing and procurement costs. However, many people compete for the same target market, and thus, the market occupied by Primary Action Group is squeezed. However, if the market is infinite, then the squeeze can be relatively ignored. Instead, many members can agree to avoid vicious price competition. Even when market space is relatively limited, such an internal agreement does not decrease business for those people in the same ethnic group; moreover, the internal standard is a steady state (i.e., not stealing business from others) and adaptive strategies (such as a career change).

After Primary Action Group perceived the high return from the union-supply model, the following quotes were provided as: “White fungus has high sales and profits and even no bargaining,” “It is still a hot product, and the union chairperson needs this food, so we offered it to him,” “Take the neighborhood committee on commission basis,”\“Run to more than 20 neighborhood committees,” “Sales are coming up,” “Selling it alone in the market,” “The quantity is not going up,” “The neighborhood committee does not have a doorperson,” “Easy to have access to,” “Give certain preferential neighborhood committees commission,” “The neighborhood committee is related and introduced to the high court,” “Meet small people first, and then, introduce them level by level,” and “Gradually form a business model, namely, trade union supply model and asset-light business model.” As CWJ stated, “Before we had to buy, I did not know how much to sell; I still have the risk of inventory and pledge funds. In addition, we are going to the wholesale market for what I want, the wholesale market sellers can earn only a little money, and we had the possibility of doubling profit.” Secondary Primary Action Groupmate, and the financial availability among clan network members is higher, the technology is easier to spread, the operation and management abilities are easier to spread, and the success rate of entrepreneurship is higher than in Primary Action Group. Members internally reduce the cost of searching for information and supervise implementation costs and bargaining decision costs, thus creating path dependence allowing entrepreneurship to enter a mature period.

Primary Action Group has close interactions with its followers in the labor market, credit markets, product markets, and other markets and has formed a stable supply chain. Some members of this group are responsible for various kinds of market procurement and joint delivery, selling products to fellow members. Moreover, some members opened factories responsible for packaging and other such tasks. At the same time as the integration of the supply chain, that of human capital also occurred, forming commercial value cocreation ability, including the internal treatment of external risks in entrepreneurship. Moreover, the reputation and brand of members are gradually becoming prominent, thus making the peer communication effect obvious. Typical quote examples and related categories are listed in Table 5.

**Table 5:** Illustrative quotations of first-order concepts in the final entrepreneurial period.

Second-order theme	First-order concept	Illustrative quotations
Entrepreneurial content design P1	Group entrepreneurship p11	“My uncle and I both went to Shanghai, and then, other villagers went to Shanghai; we all help each other.” Ap1 “At that time, my brother-in-law especially helped me purchase, we ran supply and marketing in Shanghai, as a partnership, in which everyone shared.” Bp1
	Internalized behavioral norms p12	“The family has changed from the most backward and weakest to huddled together like wolves to keep warm and achieve some achievements. The Q villagers who went to Shanghai to join CJC all work alone when they go to the local area, but we lived together, discussed business methods, sources of goods and difficulties encountered. The conclusion is unity, hard work, and the spirit of the wolf.” Bp2
Homogeneity of entrepreneurial content P2	Asset-light operation p21	“Before we were going to buy, I did not know how much I could sell, I was still at risk, and there was the danger of inventory and capital pledge, but now, it is better. In the wholesale market, we can earn only more than one yuan per pound, but there is a possibility of doubling.” Ap3
	Form business model p22	“CYS told other villagers that they could not put the stalls directly near the trade union.” Dp1 “Later, our people in the village did not have to move the stalls, directly with this model, and they took the introduction letter to state the following: ‘I want to find the trade union to sell white fungus’.” Bp2
Team behavior integration P3	Supply chain integration p31	“Market competition comes first. I am obliged to process for the family at a low cost to improve the competitiveness of the family’s products. Business is personal. As long as the family finds business, we can provide the source of goods at a low cost to help it grow and form a competitive advantage in the market.” Ep1
	Human capital integration p32	“Originally, two or three people together, one to go back to buy white fungus, and others to sell.” Fp1
Value cocreation capability P4	Internalization of external risks p41	“The business is still based on the family, and other prices cooperate with each other. If I cannot do much by myself, then I give some business to my relatives.” Gp1
	Peer-propagation effect p42	“We do not directly operate stores, but all the units are directly connected with the leaders of enterprises and institutions. We made a name for ourselves in terms of dry cargo.” Fp2

Because the relational contract contains strong personality factors, relying on the self-performance mechanism, and contains the high flexibility of contract selection, the exchange of subjects and times cannot be completely determined, allowing members to renegotiate, gain bargaining power, and increase the possibility of differences; individuals can also freely renegotiate the contract at any time (Watson et al., 2020). There is no transaction contract between villagers in Q Village, and purchasing members are only verbally informed of the goods needed. Then, those villagers who purchased the goods receive them. Even if an entrepreneur’s temporary economic situation means that he or she is unable to pay, he or she can owe the money. In this way, contract flexibility has been improved to benefit all the related parties in the cooperation process and has established a scope of adaptation to solve future conflicts through consultation.

Based on the above analysis, the paper obtains the following propositions.

Proposition C1: *In the final entrepreneurship period, under the change in external resources and environment, Primary Action Group rationally integrated the supply chain with the help of the clan to form a stable business model*.

Proposition C2: *In the final entrepreneurship period, Secondary Action Group reduced the costs of searching for information, supervision and implementation costs, and bargaining decision-making costs due to the prior information reserves and formed the value cocreation ability of the same type of business model*.

**Figure 2:**
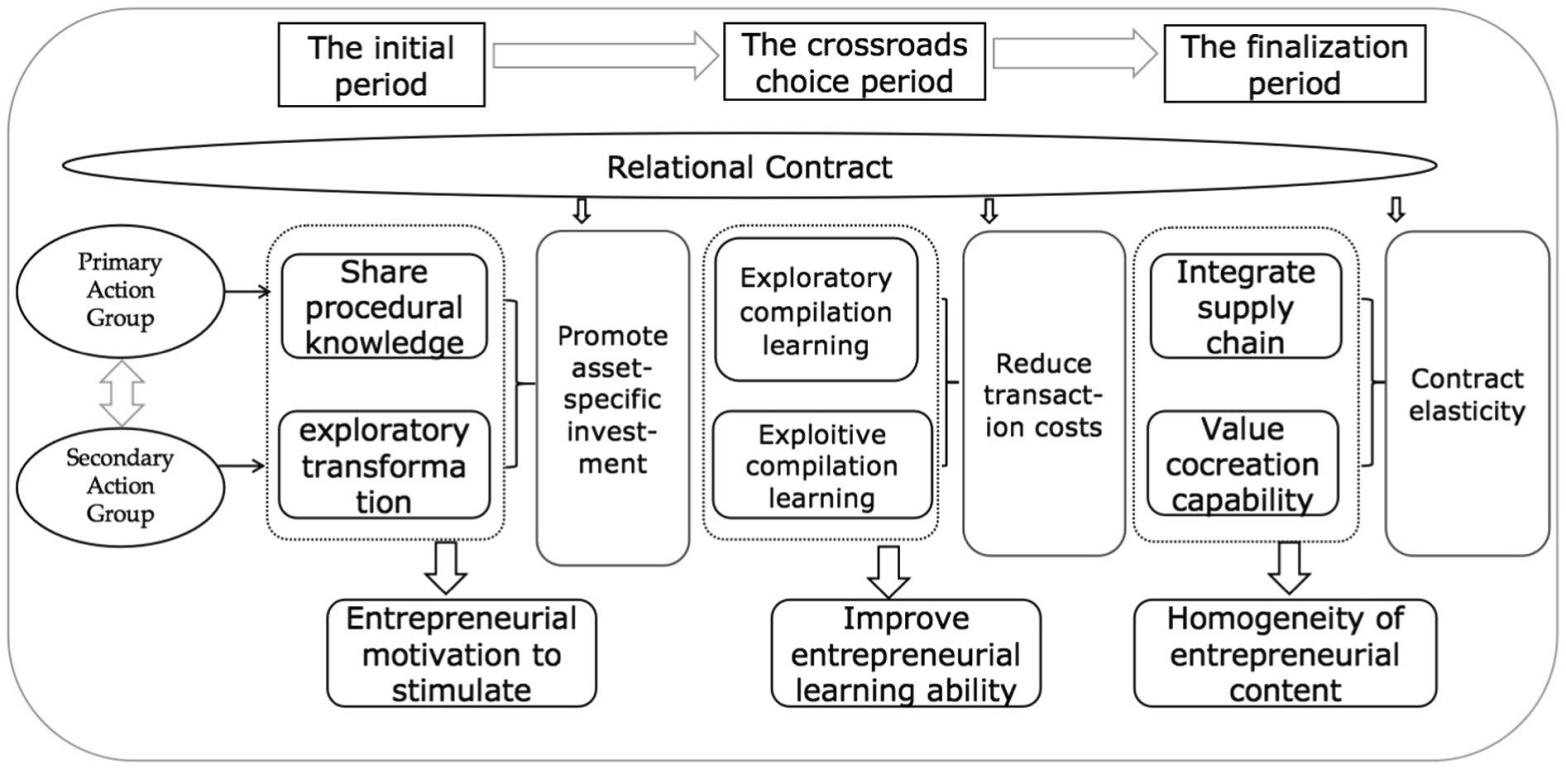
Relational contracts act on the whole process of clan member entrepreneurship.

Figure 2 refines the relational contract of the whole process of the homogeneity of clan members’ entrepreneurial business model, including each stage of different action groups, such as in the initial period of entrepreneurial motivation, in the “crossroads” period to improve members’ entrepreneurial learning ability, and in the final entrepreneurial final period when members achieve the homogeneity of their entrepreneurial business models. It should be noted that three aspects of relational contracts, namely, promoting asset-specific investment, reducing transaction costs, and releasing contract flexibility, play a role in every stage of farmers’ entrepreneurship.

The case in this paper is similar to Tan's (2020) study that most people in Xinhua of Hunan province are engaged in digital rapid printing and Wu's (2020) takes people in Sun Village of Putian, Fujian province engaged in gold and silver jewelry industry all over the country as a case study. Tan explains the mechanisms as follows: they relied on geographical relationship based on cultural intimacy of dialect much more than kinship to extend social the network. Cultural intimacy was the core mechanism for them to organize resources, reduce costs, and win in market competition. Wu finds that the emergence of “Townsman trade” is related to the resource endowment, mode of livelihood, social structure, and cultural tradition of a specific region.

We find that the relational contracts play an important role. The relational contracts are a kind of alternative arrangement of public information mechanisms. Against the background that the overall market mechanism in China is not quite perfect, the relational trust formed by both parties in the clan network plays an important role for farmers in starting businesses.

This paper analyzes the mechanism of the single-case study of Q Village and examines the key role of relational contracts in startup groups: reducing transaction costs, promoting investment in asset specificity, and improving contract flexibility. It is beneficial for different action groups to stimulate entrepreneurial motivation in the initial entrepreneurship period and improve the learning ability of entrepreneurship in the “crossroads” period. Primary Action Group transforms exploratory intuitive learning into exploratory compilation learning, and Secondary Action Group triggers the learning effect by using formula intuitive learning and using formula compiled learning, thus reducing unforeseen costs, contracting costs and verification costs, and making proprietary investments.

In the mature period, Primary Action Group rationally integrated the supply chain with the help of the clan to form a stable business model. Due to prior information reserves, Secondary Action Group reduced the costs of searching for information, supervision and execution costs, and bargaining decision-making costs and formed the value cocreation ability of the same type of entrepreneurship. To date, the completion of entrepreneurial content is homogeneous. It should be noted that the collective actions of family organizations, such as entrepreneurial actions, will gradually approach but are not necessarily equivalent to Olson’s Logic of Collective Action, where a relational contract is considered the prelude to clan collective actions.

## Conclusion and implications

The paper applies relational contract theory to explain the network structure of the clan, the external risk internalization mechanism, and the influence of farmers’ entrepreneurial business model decisions, focusing on not only the relationship between the clan network and farmers’ entrepreneurial business model but also the intermediate process of farmers’ entrepreneurial ability, expanding the boundaries of contract and entrepreneurial theory. Our results have important implications for policymakers in China and other countries with clans.

On the one hand, on the issue of grassroots government’s attitude toward clan network, considering that the relational contract formed by the clan network can promote farmers’ entrepreneurial learning and enhance their entrepreneurial ability, farmers’ entrepreneurship is still faced with practical problems such as shortage of entrepreneurial resources and insufficient entrepreneurial ability. The government can make full use of the positive role of clan network in rallying people and coordinating collective action methods to boost the prosperity of rural industries, but at the same time, it should also pay attention to curb the antagonistic clan network forces. It is suggested that regional entrepreneurship and innovation platforms should be established through organizations such as clan association to enhance farmers’ entrepreneurial ability learning and resource sharing. In the face of farmers’ entrepreneurship needs, it should give full play to the demonstration and leading ability of successful entrepreneurs within clan organizations, and actively cultivate rural entrepreneurship and innovation mentors and exert their peer group influence.

On the other hand, it should not be ignored that at present, TZTY is still in the stage of low-end development, the value added of the industry is not high, it has high substitutability, and the process of joint transformation and upgrading is slow. In the face of the development trend of branding, collectivization, and regularization, resistance to market pressure is weak, farmers easily change from “self-employment” to “becoming hired,” and entrepreneurial farmers are then squeezed out of the market.

It is suggested that local governments joint the clan network, such as clan organization integration of local characteristic resources and industry, and further integration policy, concentrated elements, cluster industry, support the development of rural entrepreneurial innovation and incubation bases, carry out internal industrial division of labor and improve the industry value chain, increase farmers’ entrepreneurship the competitiveness of the products, and build high-quality cluster development of industrial brands. Guide farmers to develop modern business management concepts, improve their entrepreneurial ability, carry out entrepreneurship and innovation through joint and in-depth cooperation, so as to promote farmers’ entrepreneurial innovation in higher quality, wider scope, and deeper fields.

## Data Availability Statement

The raw data supporting the conclusions of this article will be made available by the authors, without undue reservation.

## Ethics Statement

The informed consent of the participants was implied through survey completion. An ethics approval was not required as per applicable institutional and national guidelines and regulations.

## Author Contributions

XJ and XM: data curation. XJ, XM, and XS: methodology. XJ: writing – original draft. XS, AX, ZL, and YG: writing – review and editing. All authors contributed to the article and approved the submitted version.

## Funding

This paper was supported by the Open Foundation of Xi Jinping Thought on Socialism with Chinese Characteristics for the New Era, Minjiang University (JYLS2021003); Introduction of Talents and Social Science Project of Minjiang University (MJY21045); Fujian Province Xi Jinping Thought on Socialism with Chinese Characteristics for the New Era Research Center Project (FJ2021XZB028); Fujian Province Young and Middle-aged Teachers Education Research Project (Social Science) (JAS21263); and Fujian Social Science Foundation for Young Scholars (FJ2021C093), Fujian Innovation Strategy Research Project (2021R0092).

## Conflict of Interest

The authors declare that the research was conducted in the absence of any commercial or financial relationships that could be construed as a potential conflict of interest.

## Publisher’s Note

All claims expressed in this article are solely those of the authors and do not necessarily represent those of their affiliated organizations, or those of the publisher, the editors and the reviewers. Any product that may be evaluated in this article, or claim that may be made by its manufacturer, is not guaranteed or endorsed by the publisher.

## References

[ref1] Aghion P. Holden R. (2011). Incomplete contracts and the theory of the firm: what have we learned over the past 25 years? J. Econ. Perspect. 25, 181–197. doi: 10.1257/jep.25.2.181

[ref3] Argyres N. Bercovitz J. Zanarone G. (2020). The role of relationship scope in sustaining relational contracts in interfirm networks. Strategic. Manage 41, 222–245. doi: 10.1002/smj.3095

[ref4] Baker G. Gibbons R. Murphy K. J. (1994). Subjective performance measures in optimal incentive contracts. Q. J. Econ. 109, 1125–1156. doi: 10.2307/2118358

[ref5] Baker G. Gibbons R. Murphy K. J. (2002). Relational contracts and the theory of the firm. Q. J. Econ. 117, 39–84. doi: 10.1162/003355302753399445

[ref6] Baron R. Henry R. A. (2010). How entrepreneurs acquire the capacity to excel: insights from research on expert performance. Manage 4, 49–65. doi: 10.1002/sej.82

[ref001] Cai L. Shan B. (2013). Entrepreneurship research in The Context of China: Retrospect and Prospect. Manage. World 12, 160–169. doi: 10.19744/j.cnki.11-1235/f.2013.12.014

[ref10] Chen F. Fu L. Zhang L. Yu X. (2020). A longitudinal case study from integration perspective: how do the individual learning and organizational learning interactively influence the Firms’ innovation behaviors? Manage. World 36, 142–164. doi: 10.19744/j.cnki.11-1235/f.2020.0039

[ref11] Cruz C. Labonne J. Querubin P. (2020). Social network structures and the politics of public goods provision: evidence from the Philippines. Am. Polit. Sci. Rev. 114, 486–501. doi: 10.1017/S0003055419000789

[ref12] Davidsson P. (2015). Entrepreneurial opportunities and the entrepreneurship nexus: a re-conceptualization. J. Bus. Ventur. 30, 674–695. doi: 10.1016/j.jbusvent.2015.01.002

[ref13] Davis L. E. North D. (1992). Institutional Change and American Economic Growth. England: Cambridge University Press.

[ref14] De Wolf P. McElwee G. Schoorlemmer H. (2007). The European farm entrepreneur: A comparative perspective. Int. J. Entrep. Small Bus. 4, 679–692. doi: 10.1504/IJESB.2007.014979

[ref15] Eisenhardt K. M. (1989). Building theories from case study research. Acad. Manag. Rev. 14, 532–550. doi: 10.2307/258557

[ref16] Foltz J. Guo Y. Yao Y. (2020). Lineage networks, urban migration and income inequality: evidence from rural China. J. Comp. Econ. 48, 465–482. doi: 10.1016/j.jce.2020.03.003

[ref18] Fredenberg L Xiao Z. (2004). Game Learning Theory. China: Renmin University Press of China.

[ref19] Freedman M. (1958). Lineage Organization in Southeast China. United Kingdom: Athlone Press.

[ref20] Futterer F. Schmidt J. Heidenreich S. (2018). Effectuation or causation as the key to corporate venture success? Investigating effects of entrepreneurial behaviors on business model innovation and venture performance. Long Range Plan. 51, 64–81. doi: 10.1016/j.lrp.2017.06.008

[ref21] Gao H. (2006). Historical investigation of fellow and peers, traditional and modern— Shanghai sugar business association. Res. Chinese Eco. His. 1, 54–61. doi: 10.3969/j.issn.1000-8284.2010.08.030

[ref22] Gil R. Zanarone G. (2018). On the determinants and consequences of informal contracting. J. Econ. Manag. Sci. 27, 726–741. doi: 10.1111/jems.12246

[ref23] Goetz C. J. Scott R. E. (1981). Principles of relational contracts.Vi. Law Rev. 67.

[ref24] Greif A. Tabellini G. (2017). The clan and the corporation: sustaining cooperation in China and Europe. J. Comp. Econ. 45, 1–35. doi: 10.1016/j.jce.2016.12.003

[ref25] Guo K. (2011). Understanding of “contract team” mode: nature of contract, institutional constraints and its stakeholders. Open Times 6, 132–141.

[ref26] Han W. Yang J. Zhang Y. (2014). Case study of mixed governance mechanism selection for entrepreneurial networks. Manage. World 2, 118–136. doi: 10.19744/j.cnki.11-1235/f.2014.02.012

[ref27] Hao X. Tu Y. Chen X. Liu Y. (2019). Recalling the painful experience? Impact of emotional costs on entrepreneurial failure learning-moderating effect of counterfactual thinking. R & D Manag. 31, 27–39. doi: 10.13581/j.cnki.rdm.2019.04.004

[ref28] Johannisson B. Huse M. (2000). Recruiting Outside board members in the small family business: an ideological challenge. Entrep. & Regional Deve. 12, 353–378. doi: 10.1080/08985620050177958

[ref29] Jones M. V. Casulli L. (2014). International entrepreneurship: exploring the logic and utility of individual experience Through comparative reasoning approaches. Entrep. Theory Pract. 38, 45–69. doi: 10.1111/etap.12060

[ref002] Kinnan C. Townsend R. (2012). Kinship and financial networks, formal financial access, and risk reduction. Am. Econ. Rev. 102, 289–293. doi: 10.2307/23245544

[ref30] Kranton R. (1996). Reciprocal exchange: A self-sustaining system. Am. Econ. Rev. 86, 830–851.

[ref31] Li J. (2003). Relation-based versus rule-based governance: an explanation of the east Asian miracle and Asian crisis. Rev. Int. Econ. 11, 651–673. doi: 10.1111/1467-9396.00409

[ref32] Li X. (2013). Guangdong Business Entrepreneurship: The Power of the Family. China: Social Sciences Literature Press.

[ref33] Li Z. Huang D. J. (2021). Analysis of clans and employment in China from the aspect of gender. Growth Chang. 2, 1–25. doi: 10.1111/grow.12547

[ref34] Liu S. (1999). Embedding and relational contract. Sociological. Aust. Stud. 4, 77–90.

[ref35] Liu S. (2003). Possession, Cognition and Interpersonal Relational: Economic and Sociological Analysis of Rural Institutional Changes in China: Huaxia Publishing House.

[ref36] Luo J. (2011). The market power of the circle culture. Bus. Commun. 5, 40–44.

[ref37] Luo B. (2017). Contract short term with empty contract hypothesis —— based on farm lease empirical evidence. Res. Fin. Issues 1, 10–21. doi: 10.3969/j.issn.1000-176X.2017.01.002

[ref39] Ma G. Yang E. (2011). Social networking. Irfo. Fin. Ent. Econ. Research 3, 83–94.

[ref40] Martins L. L. Rindova V. P. Greenbaum B. E. (2015). Unlocking the hidden value of concepts: a cognitive approach to business model innovation. Strateg. Manag. J. 9, 99–117. doi: 10.1002/sej.1191

[ref41] Michler J. D. Wu S. Y. (2020). Relational contracts in agriculture: theory and evidence. Ann. Rev. Resour. Econ. 12, 111–127. doi: 10.1146/annurev-resource-101719-034514

[ref43] Naminse E. Y. Zhuang J. Zhu F. (2019). The relation between entrepreneurship and rural poverty alleviation in China. Manag. Decis. 57, 2593–2611. doi: 10.1108/MD-11-2017-1153

[ref44] Politis D. (2005). The process of entrepreneurial learning: a conceptual framework. Entrep. Theory Pract. 29, 399–424. doi: 10.1111/j.1540-6520.2005.00091.x

[ref003] Shepherd D. A Patzelt H. (2018). Entrepreneurial Cognition: Exploring the Mindset of Entrepreneurs. Palgrave Macmillan: Springer International Publishing.

[ref46] Sun A. (2013). Relational contract governance between enterprises. Contem. Econ. Research 11, 30–34.

[ref47] Sun J. Li Y. (2020). Mobile handicraft society: from “peer industry” to “regional industry” of Bai silver Instrument Village. Open Time 4, 57–69+6.

[ref48] Sun Y. Yu M. (2010). Review on relational contract theory. Acad. Exch. 8, 117–123. doi: 10.3969/j.issn.1000-8284.2010.08.030

[ref49] Tan T. (2012). Kininity, geography and market. Open Times 6, 69–81.

[ref50] Tan T. (2020). Limited differential mode of association and its modern transition: A reflection on printing industry run by Xinhua people. J. Nanjing Agri. Univ. 20, 18–28. doi: 10.19714/j.cnki.1671-7465.2020.0072

[ref51] Tseng C. (2013). Connecting self-directed learning with entrepreneurial learning to entrepreneurial performance. Int. J. Entrep. Behav. R. 19, 425–446. doi: 10.1108/IJEBR-08-2011-0086

[ref52] Uzzi B. (1997). Social structure and competition in Interfirm networks: The paradox of Embeddedness. Adm. Sci. Q. 42, 35–67. doi: 10.2307/2393808

[ref53] Wang Y. (2006). Interlinking markets, relational contract and economic transition. Econ. R. 6, 79–91.

[ref004] Wang Y. (2019). A pyramid model of successor’s entrepreneurial growth in family firms: multi-case study from the perspective of meaning making. Manage. World 35, 168–184+200. doi: 10.19744/j.cnki.11-1235/f.2019.0027

[ref55] Wang Y. Li M. (2008). Understanding China’s economic miracle: an internet contract perspective. Manage. World 10, 5–20+187. doi: 10.19744/j.cnki.11-1235/f.2008.10.002

[ref56] Watson J. Miller D. A. Olsen T. E. (2020). Relational contracting, negotiation, and external enforcement. Am. Econ. Rev. 110, 2153–2197. doi: 10.1257/aer.20180427

[ref8] Welch C. E. Piekkari R. Plakoyiannaki E. Paavilainen-Mäntymäki E. (2011). Theorising from case studies: towards a pluralist future for international business research. J. Int. Bus. Stud. 42, 740–762. doi: 10.1057/jibs.2010.55

[ref57] Williamson O. E. (1996).The Mechanisms of Governance. England: Oxford University Press.

[ref58] Wu C. (2020). “Fellow townsman”: “social economy” or “low-end nationalization”? J. Nanjing Agri. Univ. 20, 9–17. doi: 10.19714/j.cnki.1671-7465.2020.0071

[ref59] Yang G. (2007). From the “relational contract” to the “institutionalized cooperation”: the evolution path of the internal cooperation mechanism of the private chamber of commerce-takes the Wenzhou chamber of commerce as an example. China Adm. 8, 37–40.

[ref60] Yang H. (2019). Family clans and public goods: evidence from the New Village beautification project in South Korea. J. Dev. Econ. 136, 34–50. doi: 10.1016/j.jdeveco.2018.09.001

[ref62] Yu W. Choi M. Zheng J. (2021). How do different types of entrepreneurial networks and decision-making influence the identification of entrepreneurial opportunities? Front. Psychol. 12:683285. doi: 10.3389/fpsyg.2021.683285, PMID: 34335396 PMC8322445

[ref63] Zhang C. (2020). Clans, entrepreneurship, and development of the private sector in China. J. Comp. Econ. 48, 100–123. doi: 10.1016/j.jce.2019.08.008

[ref64] Zhang Y. Feng X. (2019). Academic problems and research suggestions driven by the entrepreneurial practice of agriculture, rural areas and farmers. Southern Econ. 7, 72–82. doi: 10.19592/j.cnki.scje.361901

[ref65] Zheng L. (2014). Traditional of southeast Asian Chinese takes Furong po Xinghua as an example. Open. Time 1, 210–223+9. doi: 10.3969/j.issn.1004-2938.2014.01.010

